# Inductive Displacement Sensors with a Notch Filter for an Active Magnetic Bearing System

**DOI:** 10.3390/s140712640

**Published:** 2014-07-15

**Authors:** Seng-Chi Chen, Dinh-Kha Le, Van-Sum Nguyen

**Affiliations:** Department of Electrical Engineering, Da-Yeh University, Changhua 51591, Taiwan; E-Mails: ledinhkha@yahoo.com (D.-K.L.); nvsum@hueic.edu.vn (V.-S.N.)

**Keywords:** displacement measurement, notch filter, natural frequency, inductive displacement sensors, lock-in amplifier, AMB

## Abstract

Active magnetic bearing (AMB) systems support rotating shafts without any physical contact, using electromagnetic forces. Each radial AMB uses two pairs of electromagnets at opposite sides of the rotor. This allows the rotor to float in the air gap, and the machine to operate without frictional losses. In active magnetic suspension, displacement sensors are necessary to detect the radial and axial movement of the suspended object. In a high-speed rotating machine equipped with an AMB, the rotor bending modes may be limited to the operating range. The natural frequencies of the rotor can cause instability. Thus, notch filters are a useful circuit for stabilizing the system. In addition, commercial displacement sensors are sometimes not suitable for AMB design, and cannot filter the noise caused by the natural frequencies of rotor. Hence, implementing displacement sensors based on the AMB structure is necessary to eliminate noises caused by natural frequency disturbances. The displacement sensor must be highly sensitive in the desired working range, and also exhibit a low interference noise, high stability, and low cost. In this study, we used the differential inductive sensor head and lock-in amplifier for synchronous demodulation. In addition, an active low-pass filter and a notch filter were used to eliminate disturbances, which caused by natural frequencies. As a consequence, the inductive displacement sensor achieved satisfactory linearity, high sensitivity, and disturbance elimination. This sensor can be easily produced for AMB applications. A prototype of these displacement sensors was built and tested.

## 1. Introduction

In active magnetic bearing (AMB) systems, the equilibrium point of the rotor shaft is always located in the center of the stator. The air gap between the rotor and AMB stator is small; normally it is smaller than 0.5 mm. Displacement sensors are a part of magnetic bearing systems. All displacement sensors are used, and must be able to perform measurements on a rotating surface. A rough surface, geometrical errors, the sampling rate, and natural frequencies of the rotor may cause noise disturbances; thus, an active low-pass filter and notch filter must be fitted to the sensor [1–3].

Displacement sensors detect the linear position during the movement of a shaft without mechanical contact. Three basic types of displacement sensor exist: capacitive, laser, and electromagnetic sensors. In capacitive sensors, the air gap length is detected based on variations in capacitance. Therefore, satisfactory isolation between the sensor and the shaft is required, because it can change the dielectric. In laser sensors, displacements are detected by using a reflected laser light, thus, a uniform target surface is required to prevent noise [2]. In some cases, these sensors can be used in AMB system applications; however, electromagnetic sensors are considered to be effective for general purpose applications. Two types of electromagnetic sensors exist: inductive and eddy-current type. In inductive sensors, the target is made of a ferromagnetic material with high permeability, such as laminated silicon steel, ferrite, or carbon steel. In eddy-current sensors, the target is made of a conductive material such as copper, non-magnetic stainless steel, aluminum, carbon steel, or other metallic materials. Depending on the application of the magnetic bearing and mechanical factors, a sensor suitable for each application can be designed [2–5].

Furthermore, the natural frequencies and bending modes of the rotor at a high rotation speed are critical [6]. The collocation of each bearing units and displacement sensors is a common practice in industrial AMB systems to decentralize control scheme [1,2]. Thus, the values of the displacement sensor output must be accurate and noise must be avoided. The notch filter is used to remove the frequency noise causing by the rotor dynamics. In addition, general functions of displacement sensor in commercial industry do not considerate with natural frequencies and bending modes. Hence, implementing displacement sensors with an AMB structure combining notch filter functions is useful in AMB systems [4,5].

In this study, we implemented inductive displacement sensors using a differential inductive sensor head, lock-in amplifier, and filters. The DC voltage output of the inductive sensor is linearity [7]. Lock-in amplifiers are used to detect and measure a small displacement of the rotor. It uses a technique known as phase-sensitive detection (PSD) to identify a component of the signal at a specific reference frequency and phase. Noise signals whose frequencies differ from the reference frequency are rejected, thus they do not affect the measurement. This technique is called synchronous demodulation [8].

The following points are discussed:

(1) The AMB system structure.
(2) Calculation of rotor resonances.
(3) Inductive sensor head.
(4) Lock-in amplifier or synchronous demodulation.
(5) Low-pass filter and notch filter.
(6) Built inductive sensors using IC AD630 and test rig results.

## 2. The Magnetic Bearing Construction System and Natural Frequencies of Rotor

**Figure 1.**
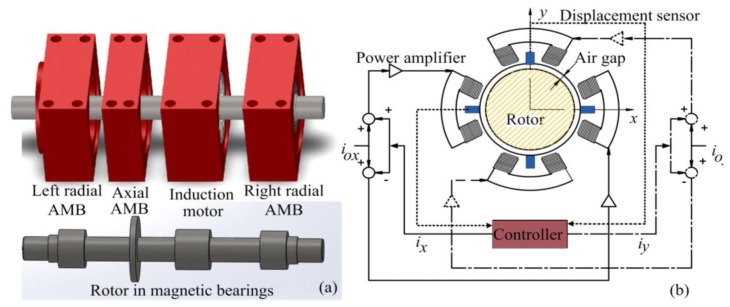
(**a**) Horizontal shaft—Active magnetic bearing (AMB) structure; (**b**) Radial AMB with current control.

Figure 1a shows the typical structure of a motor drive system, using magnetic bearings, comprising a left radial AMB, right radial AMB, axial AMB, and an induction motor. Each radial magnetic bearing generates radial forces along two perpendicular radial axes. The radial forces are controlled, so that the shaft position is regulated in the center of the stator at the equilibrium position. Figure 1b shows the technical application of the AMB. For motion of the two degrees of freedom, two opposing electromagnets are operated in the differential driving mode [1,2]. In this configuration, the electromagnetic force is exerted on the rotor in arbitrary directions of the *x* axis and *y* axis to maintain the rotor in the center. *i*_0_*_x_* and *i*_0_*_y_* are bias currents, and *i_x_*, *i_y_* are control currents along the *x* and *y* axes, respectively. Positioning the rotor requires two differential displacement sensors to measure the displacement value following the *x* and *y* axes [3]. AMBs suspend a rotor with feedback control through a measured displacement sensor signal. The qualities of a control system depend on the quality of the sensor signal, particularly in precision applications [9].

Furthermore, accurately computing the natural frequencies of the rotor is crucial when it rotates at high speed. At natural frequencies, the rotor vibrates considerably. Since the displacement sensor can filter the consonance frequencies of the rotor, the quality of non-linear controllers will be improved [6].

**Figure 2.**
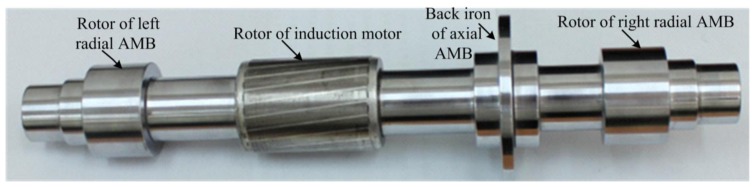
Rotor inside AMB.

The rotor, shown in Figure 2, consists of a steel shaft with a radial AMB end-cap at both ends, and in the middle, it contains the rotor of induction motor and axial AMB.

Initially, a circular steel shaft, machined from AISI 4340 steel, was considered. This steel shaft had a 346 mm axial length and a mass of 3.42 kg. AISI 4340 steel is a magnetic stainless steel, and its specifications are listed in Table 1.

In this work, the natural frequencies are estimated by analytical equation and are simulated by ANSYS software.

Firstly, the shaft's natural frequencies were calculated using Equation (1), as follows [5]:

(1) $$\omega_{n}=a_{n}\sqrt{\frac{EI}{\mu_{1}l^{4}}}$$

where *E*, *I*, *μ*_1_, and *l* are the Young's modulus, inertia, mass per unit length, and length of the shaft, respectively, and *a_n_* is a numerical constant calculated by using the Rayleigh method [5], which depends on the problem boundaries. The values of *a_n_* from the first to the third free of bending modes follow the bending vibrations of “free-free” beam constants: *a*_1_ = 22, *a*_2_ = 61.7, *a*_3_ = 121.

**Figure 3.**
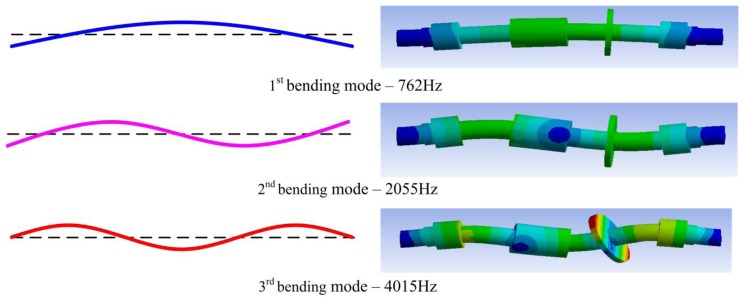
Shaft bending mode.

Using Equation (1), we calculated the natural frequencies of the circular rotor in Table 2:

Secondly, the rotor is modelled and simulated by ANSYS, and then the natural frequencies can be determined. The natural frequencies are listed in Table 3. The simulation results of rotor bending mode are shown in Figure 3. The results indicate that the values of the natural frequencies estimated by the two approaches are close together [6].

## 3. Inductive Sensor Head

Inductive proximity sensors operate under the electrical principle of inductance. An inductor coil on a ferromagnetic core is driven by an oscillator. When a ferromagnetic object approaches the coil of an inductor, the inductance is changed. The inductance variation is proportional to the displacement of the ferromagnetic object [7]. This change in inductance is sensed by the electronics function board, and converted to a sensor output voltage proportional to the displacement. The two sensor heads oppose each other, and the differential position is frequently arranged on a rotor. These sensor heads are operated in a differential mode in a Wheatstone bridge circuit with a constant AC signal [10,11].

**Figure 4.**
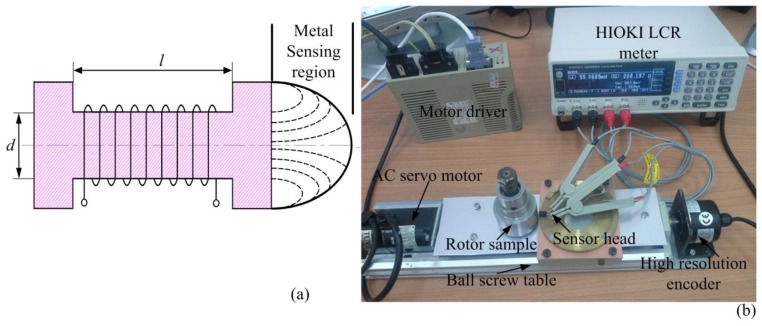
(**a**) Inductive sensor head structure; (**b**) Sensor head characteristic experiment.

Figure 4a displays the inductive sensor head structure, an inductance *L* can be express follows:

(2) $$L=\frac{\mu N^{2}A}{l}(\text{H})$$

where

*N* = number of turns of the coil.
*μ* = effective permeability of the medium.
$A=\frac{\pi d^{2}}{4}$, area of the core (m^2^), *l* = length of the coil and *d* = diameter of the core.

**Figure 5.**
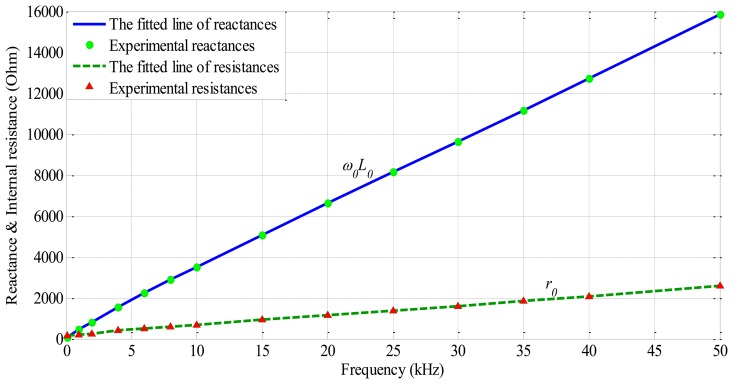
Frequency characteristic of inductive sensor head.

**Figure 6.**
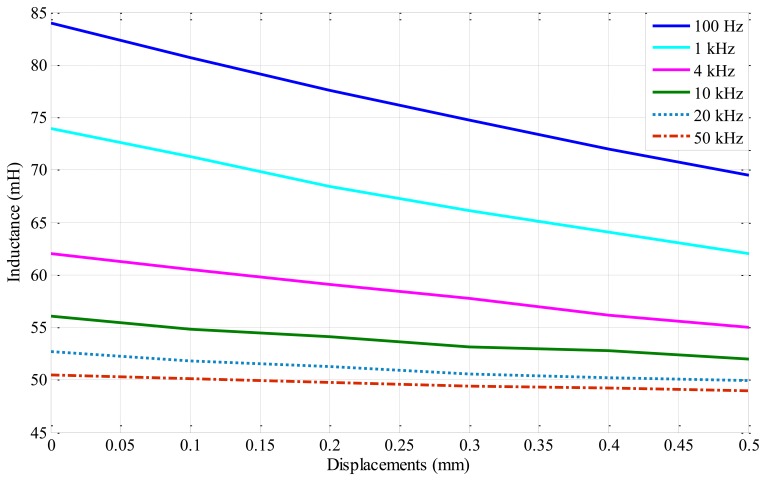
Characteristic of the inductance *versus* the displacement.

**Figure 7.**
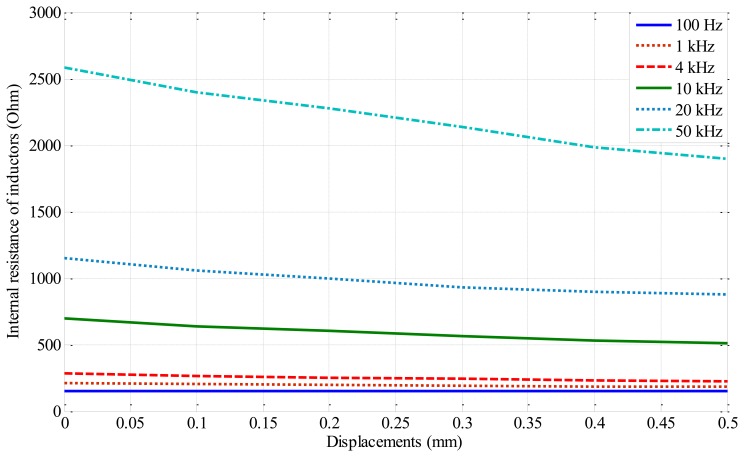
Characteristic of the internal resistance *versus* the displacement.

A sensor measurement system based on a commercially available LCR meter and a test rig with high precision servo mechanisms, which is shown in Figure 4b, experiment sensor head with ferrite core, *N* = 1300, *d* = 2.5 mm, *l* = 4.5 mm, the frequency and displacement characteristics were measured in Figures 5, 6 and 7. In Figure 5, the internal resistance of sensor head is sufficiently small in comparison with its reactance.

In Figure 6 illustrates that when increase exciting frequency excites to the inductance of sensor head in the range of displacement from 0 to 0.5 mm, the sensitive inductance decrease.

In addition, in Figure 7 illustrates that increasing the exciting frequency to the inductive sensor head, the internal resistance of inductor coil also increase. This value affects the linearity of DC voltage output.

Implementing the inductive displacement sensor with high sensitive, the frequency of AC excitement must be set in range from 5 kHz up to 100 kHz prevent eddy current obstructing the formation of magnetic path on the surface of rotor and the resistance of the sensor should be sufficiently small in comparison with its reactance [1]. In this experiment, with sampling frequency for xPC real time target is 4 kHz, we used a modulation frequency of 10 kHz, and the cut-off frequency was 4 kHz, which is sufficient to control an AMB system.

**Figure 8.**
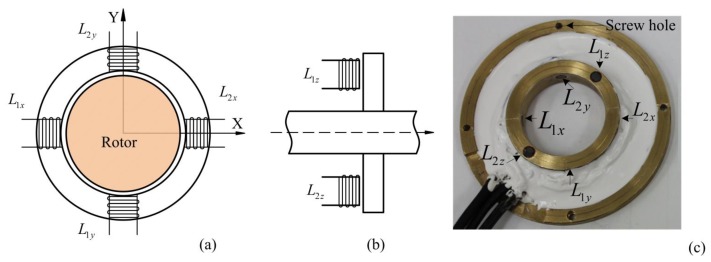
Inductive sensor head position, (**a**) radial sensors; (**b**) axial sensors; (**c**) experimental setup.

For measuring the sensitive displacement of the rotor in an air gap, we used a differential inductive sensor head, which is shown in Figure 8.

**Figure 9.**
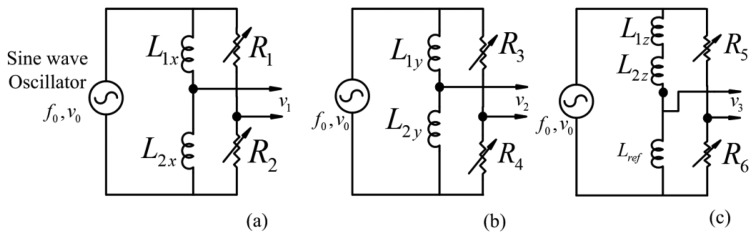
The Wheatstone bridges equivalent diagram, (**a**) *x* axis differential inductive sensor; (**b**) *y* axis differential inductive sensor; (**c**) *z* axis differential inductive sensor.

Figure 8 illustrates the basic structure of inductive sensors. The inductive sensor heads, placed opposite each other, form as differential circuit. Figure 8a displays the positions of the inductive sensor heads *L*_1_*_x_*, *L*_2_*_x_*, *L*_1_*_y_*, *L*_2_*_y_*, in the radial sensor housing and the in Figure 8b inductive sensor heads *L*_1_*_z_*, *L*_2_*_z_*, in the axial sensor housing. Figure 9a,b illustrates the Wheatstone bridges equivalent diagram of sensor heads for radial sensors. As shown in Figure 9c, the axial inductive sensor head *L*_1_*_z_*, and *L*_2_*_z_*, are placed on the same side, thus, the external reference inductor with the *L_ref_* inductance is equal to sum of *L*_1_*_z_*, *L*_2_*_z_*, and then is combined with two variable resistors as the Wheatstone bridge [12].

The voltage *v*_1_, in Figure 9a, between the middle points of the sensor coils and the external resistors is expressed as follow:

(3) $$v_{1}=\left(\frac{R_{1}}{R_{1}+R_{2}}-\frac{r_{1}+j\omega_{0}L_{1}}{r_{1}+j\omega_{0}L_{1}+r_{2}+j\omega_{0}L_{2}}\right)v_{0}$$

where, *f*_0_ is the frequency of AC excitement oscillator and *ω*_0_ = 2*πf*_0_, *R*_1_ and *R*_2_ are resistances of the external resistor, *L*_1_ and *L*_2_ are equivalent inductances of the two sensor coils *L*_1_*_x_* and *L*_2_*_x_*, and *r*_1_, *r*_2_ are internal resistances of *L*_1_*_x_*, *L*_2x_.

When the rotor is at the center position of the AMB, *R*_1_ = *R*_2_ = *R*_0_, *r*_1_ = *r*_2_ = *r*_0_ and the sensor inductance is *L*_0_.

When the rotor displacement Δ*x* in an air gap: *L*_1_ = *L*_0_ + *L*_x_ and *L*_2_ = *L*_0_ − *L_x_*.

*v*_1_ is given as:

(4) $$v_{1}=\frac{v_{0}}{2}L_{x}\;\left(\frac{\omega_{0}^{2}L_{0}}{\omega_{0}^{2}L_{0}^{2}+r_{0}^{2}}\right)+j\frac{v_{0}}{2}L_{x}\;\left(\frac{\omega_{0}L_{0}}{\omega_{0}^{2}L_{0}^{2}+r_{0}^{2}}\right)$$

In Equation (4), when the sensor head changes Δ*x* in air gap length, the phase difference is dependent on the Δ*x* displacement.

*v*_1_ and *v*_0_ in Equation (4) connect to lock-in amplifier used to detect the amplitude and differential phase proportional to the displacement.

## 4. Lock-In Amplifier

Lock-in amplifiers are used to detect and measure small AC signals. Accurate measurements may be achieved even when the small signal is obscured by a noise source many thousands of times larger. Lock-in amplifiers use a technique known as PSD to identify a component of the signal at a specific reference and phase [8]. Noise signals, at frequencies other than the reference frequency are rejected and do not affect the measurement. In AMB system, we used a lock-in amplifier with PSD and filters, thereby eliminating most of the broadband noise. It enhances the signal to noise ratio (SNR) by averaging the measurement. In this case the noise goes down as the square root of the number of averaged samples [11].

**Figure 10.**
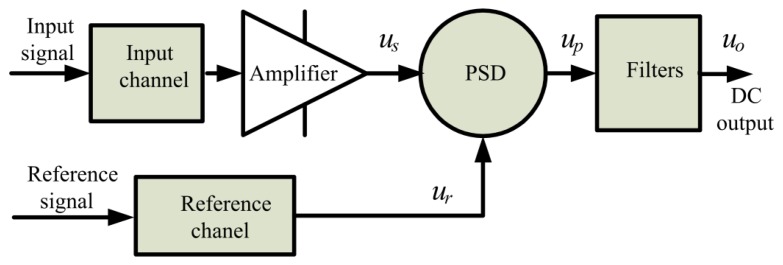
Lock-in amplifier block diagram.

A common basic structure of lock-in amplifiers comprising a signal channel, reference channel, PSD, low-pass, and notch filters is shown in Figure 10.

PSD is the core of the lock-in amplifier. The output depends not only on the magnitude of the input signal, but also on the phase between the reference signal and the input signal. PSD is commonly used in analog multipliers and electronic switching, the output is as follows:

(5) $$u_{p}=u_{s}u_{r}$$

Suppose the reference signal is *u_r_* = *U_r_* sin*ωt*, input signal is *u_s_* = *U_s_* sin(*ωt* + *φ*) and *φ* is the phase difference. Using Equation (5), the following expression can be obtained:

(6) $$u_{p}=\frac{U_{s}U_{r}}{2}\cos\varphi -\frac{U_{s}U_{r}}{2}\cos\left(2\omega t+\varphi \right)$$

Equation (6) reveals that the first term is the difference frequency, and the second term is the sum frequency. Through a low-pass filter, the sum frequency is filtered, and the result is expressed as:

(7) $$u_{0}=\frac{U_{s}U_{r}}{2}\cos\varphi$$

Equation (7) shows that the lock-in amplifier output signal is only related to amplitudes of the input signal, amplitudes of the reference signal and the phase difference between the reference signal and the input signal.

## 5. Active Low-Pass Filter and Notch Filter

### 5.1. The Active Low-Pass Filter

**Figure 11.**
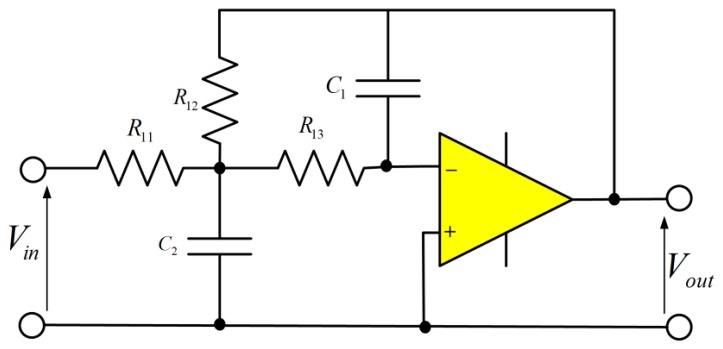
Active low-pass filter diagram.

An active low-pass filter allows low frequency signals to pass from input to output, while attenuating the high frequency signals. The active low-pass filter is illustrated in Figure 11. The active filter uses an operational amplifier in addition to the resistors and capacitors [13].

The transfer function is as follows:

(8) $$\frac{V_{\text{out}}}{V_{in}}=-\frac{\frac{1}{R_{11}}\frac{1}{R_{13}}}{C_{1}s\;\left(\frac{1}{R_{11}}+C_{2}s+\frac{1}{R_{12}}+\frac{1}{R_{13}}\right)+\frac{1}{R_{12}}\frac{1}{R_{13}}}$$

Rearranging yields:

(9) $$\frac{V_{\text{out}}}{V_{in}}=-\frac{\frac{1}{R_{11}R_{13}}}{s^{2}C_{1}C_{2}+sC_{1}\;\left(\frac{1}{R_{11}}+\frac{1}{R_{12}}+\frac{1}{R_{13}}\right)+\frac{1}{R_{12}R_{13}}}$$

Dividing by *C*_1_*C*_2_, we have the standard form:

(10) $$\frac{V_{\text{out}}}{V_{in}}=-\frac{\frac{1}{R_{11}R_{13}C_{1}C_{2}}}{s^{2}+s\frac{1}{C_{2}}\;\left(\frac{1}{R_{11}}+\frac{1}{R_{12}}+\frac{1}{R_{13}}\right)+\frac{1}{R_{12}R_{13}C_{1}C_{2}}}$$

The transfer function is:

(11) $$TF=\frac{A_{0}\omega_{0}^{2}}{s^{2}+\frac{\omega_{0}}{Q}s+\omega_{0}^{2}}$$

where $\omega_{0}^{2}=\frac{1}{R_{12}R_{13}C_{1}C_{2}}$ and therefore, the low-pass cut-off frequency

(12) $$\omega_{0}=\sqrt{\frac{1}{R_{12}R_{13}C_{1}C_{2}}}$$

as well as $\frac{\omega_{0}}{Q}=\frac{1}{C_{2}}\left(\frac{1}{R_{11}}+\frac{1}{R_{12}}+\frac{1}{R_{13}}\right)$, and therefore the quality factor,

(13) $$Q=\frac{\sqrt{\frac{C_{2}}{C_{1}R_{12}R_{13}}}}{\frac{1}{R_{11}}+\frac{1}{R_{12}}+\frac{1}{R_{13}}}$$

The gain at zero frequency is as follows:

(14) $$A_{0}=\frac{V_{\text{out}}}{V_{in}}=-\frac{R_{12}}{R_{11}}$$

**Figure 12.**
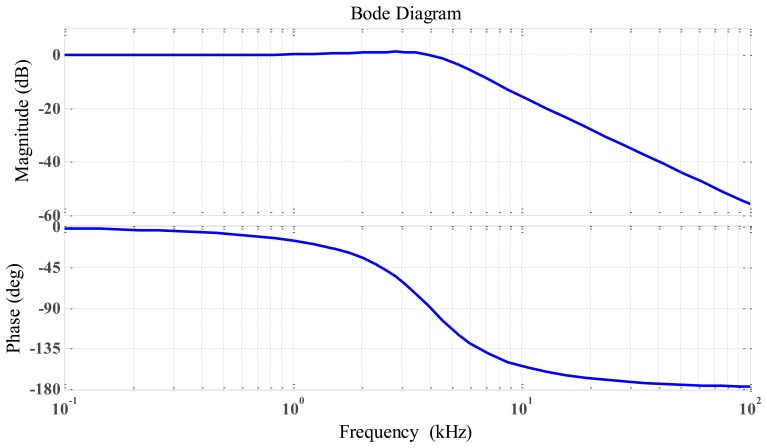
Frequency response for active low-pass filter using operational-amplifier.

The resistor and capacitor were calculated using a cut-off frequency *ω*_0_ of 4 kHz, the frequency response for an active low-pass filter using operational amplifier illustrated in Figure 12.

### 5.2 Notch Filter

**Figure 13.**
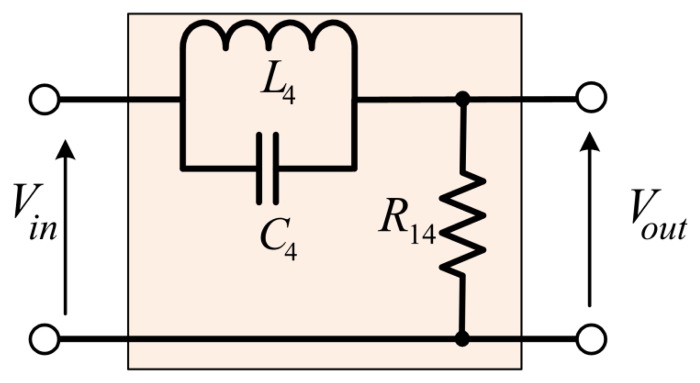
Notch filter diagram.

The notch filter allows all frequencies to pass, with the exception of a narrow band which is greatly attenuated. In the analog design, the notch filter generally uses LC resonance to eliminate the unexpected frequency [13]. A notch filter comprising of resistor, inductor, and capacitor is shown in Figure 13.

The transfer function:

(15) $$TF=\frac{R_{14}}{R+Z_{L_{4}}||Z_{C_{4}}}=\frac{1-\omega^{2}L_{4}C_{4}}{1-\omega^{2}L_{4}C_{4}+j\omega \frac{L_{4}}{R_{14}}}$$

The transfer function in Equation (15) was rewrite using Laplacian *s* in place *jω*:

(16) $$TF=\frac{s^{2}+\omega_{n}^{2}}{s^{2}+\frac{\omega_{n}}{Q}s+\omega_{n}^{2}}$$

where, the notch frequency $\omega_{n}=\sqrt{\frac{1}{L_{4}C_{4}}}$ and the quality factor is $Q=\frac{R_{14}}{L_{4}}$.

The notch filter circuit effectively removes any signal occurring at the resonant frequency. For low frequency signals, the inductor provides a low impedance path from input to output, allowing these signals to pass from the input, and appear across the resistor with minimal attenuation. Conversely, at high frequencies, the capacitor provides a low impedance path from input to output.

**Figure 14.**
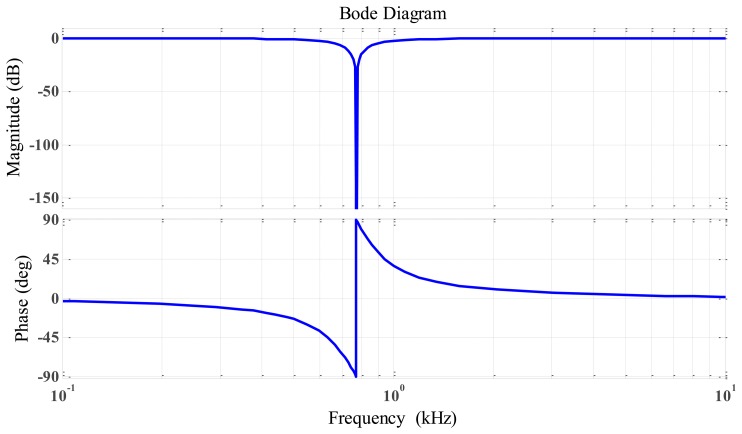
Frequency response for RLC notch filter.

As obvious listed in Table 2, the rotation speed was set at 50,000 rpm higher than the first bending mode at 46,200 rpm or 770 Hz, thus, we used only one notch filter circuit with notch frequency = 770 Hz. The resistor and capacitor values were calculated, and the frequency response for the RLC notch filter is shown in Figure 14.

## 6. Implementing an Inductive Displacement Sensor

### 6.1. Block and Schematic Design

**Figure 15.**
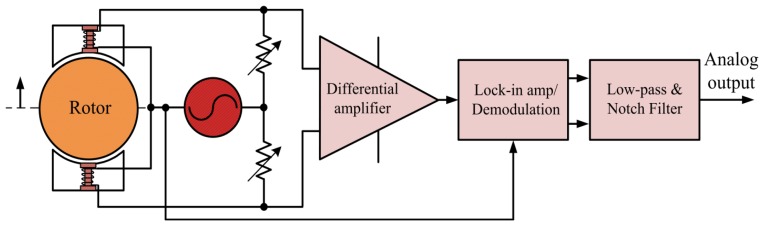
The inductive displacement sensor block diagram.

**Figure 16.**
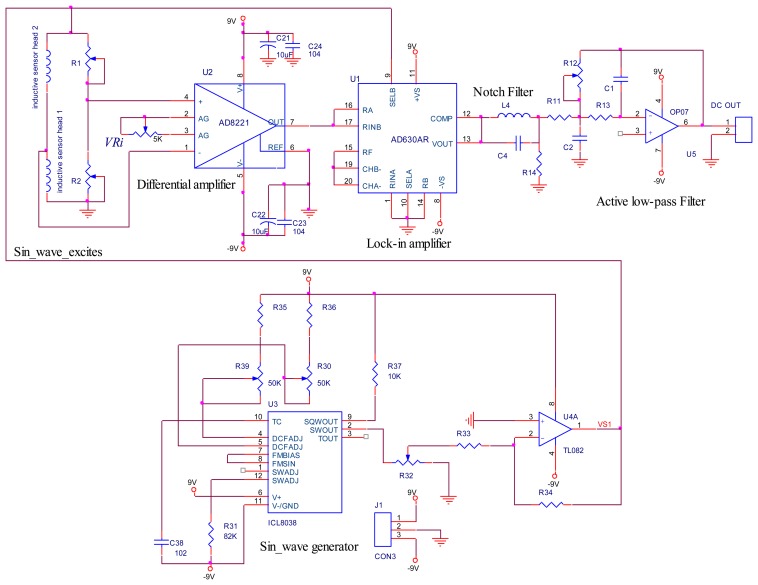
Full Schematic of inductive displacement sensor for AMB.

Figure 15 displays the inductive displacement sensor block diagram. As the schematic design in Figure 16 shows, the ICL8038 waveform generator was used for accuracy sine wave generation with frequency 10 kHz. Another IC, the AD630, is a high precision balanced modulator, its signal processing applications include PSD, lock-in amplification, synchronous demodulation [14,15].

A 10 kHz sine wave signal exciting the Wheatstone bridge was generated by ICL8038. The signal input in the AD8221 was proportional to the displacement of the rotor, according to Equation (4). By changing the variable resistor *VRi* value at Pin 2 and Pin 3 of AD8221, we changed the gain of AD8221, which can be calculated using the following gain equation.

(17) $$\mathrm{VRi}=\frac{49.4k\Omega }{\text{Gain}-1}$$

The output signals from AD630 travel to the notch filter, then to the active low pass filter, and the output is the DC voltage. We can change the gain of the active low-pass filter by calibrating the variable resistor R12.

**Figure 17.**
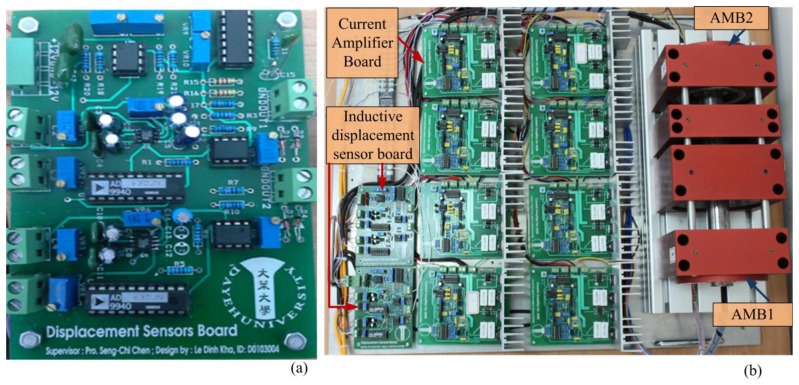
(**a**) Experimental layout board; (**b**) AMB test rig functions.

Figure 17a displays the inductive displacement sensor board, and Figure 17b displays the AMB test rig functions. In this board, we used one chip sine wave generator ICL8038 chip and two AD630 chips corresponding to an *x* displacement sensor and *y* displacement sensor [14,15].

### 6.2. PSpice Simulation

**Figure 18.**
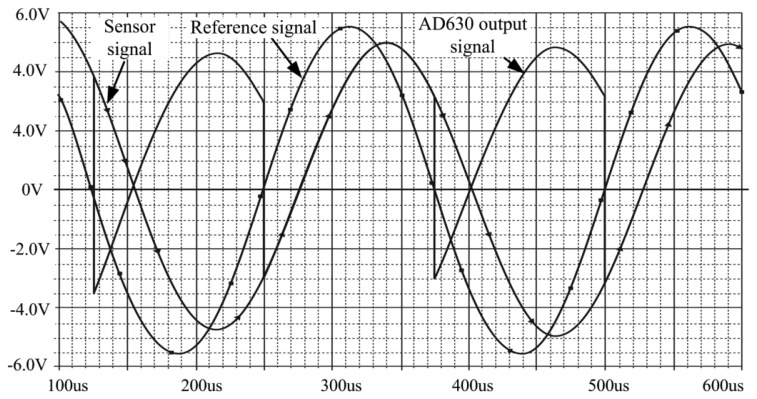
Pspice simulation result.

This design was also simulated using the PSpice software. The result is illustrated in Figure 18. The output signal changed follows the phase shift of the reference signal and sensor input signal.

### 6.3. Experimental Results

**Figure 19.**
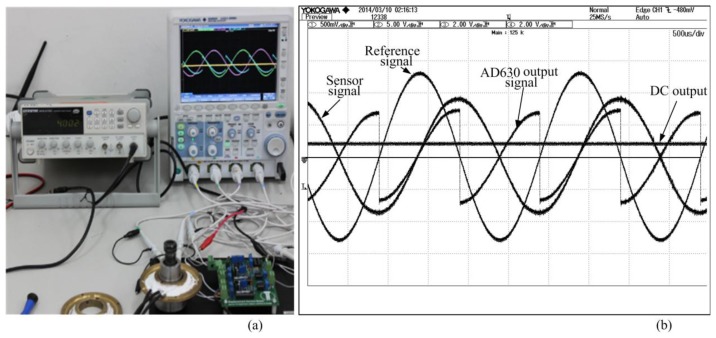
Experimental result in oscilloscope, (**a**) Experimental board setup; (**b**) Signal measurements.

We used a differential inductive sensor and lock-in amplifier, the output signal was shown in Figure 19.

**Figure 20.**
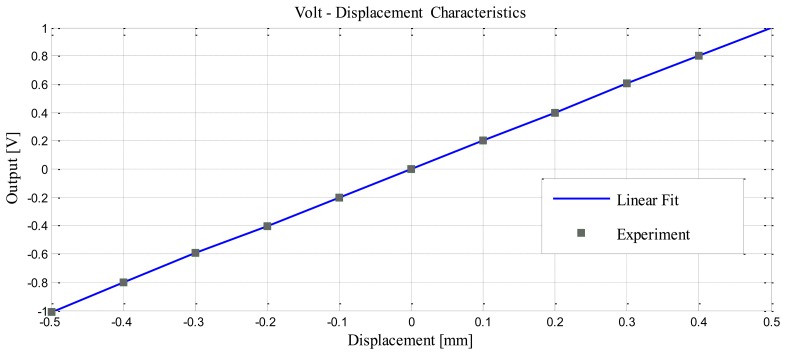
Volt-Displacement (mm) characteristic.

**Figure 21.**
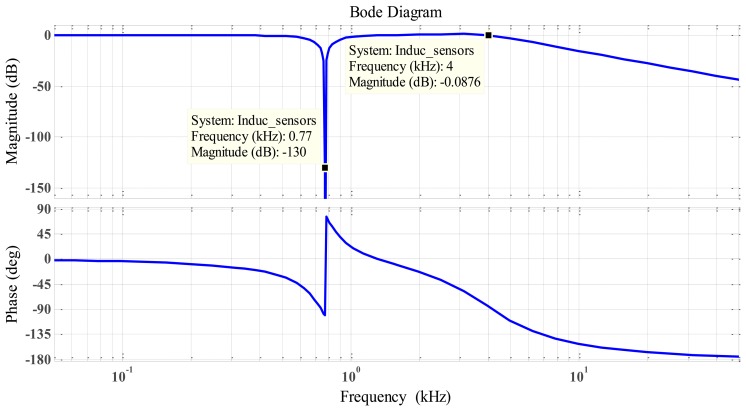
Frequency response of inductive displacement sensor exhibiting notch filter.

Figure 20 shows that the DC output was linear. We can change the slope output by changing the gain of the active low-pass filter by the way changing the value of R12. Figure 21 illustrates the response frequency of this sensor. The cut-off frequency was 4 kHz, and notch frequency was 770 Hz, The specification results of the differential inductive displacement sensor are summarized in Table 4.

## 7. Conclusion and Outlook

Displacement sensors detect linear positions during the movement of a shaft without mechanical contact. The sensor is a key component, necessary to determine the quality of non-linear controller in an AMB system. Sensor performance directly affects the precision control of the entire system.

In this paper, an inductive displacement sensor has been developed and evaluated. The sensor possesses high linearity and sensitive characteristics by integrating a lock-in amplifier, an active low-pass filter, and a notch filter. In addition, when applying for an AMB system, this architecture of the sensor allows eliminating noises generated by natural frequencies of the rotor. We have also shown two kind of inductive displacement sensors, axial and radial ones. The two sensors can be combined into one sensor housing. As a result, the sensor is suitable for industrial AMB systems where decentralized control scheme was applied. Overall, the performance of the inductive displacement sensor can meet the practical requirements of an AMB system.

In an AMB system, the rotors are normally coated with thin Chromium material. Hence, the affecting of a thin Chromium coated rotor to the sensitivity of the sensor is further investigation.

## Figures and Tables

**Table 1.** Stainless steel AISI4340 specifications.

**Density (*ρ*)**	**7850 kg/m^3^**
Young's Modulus (*E_x_*, *E_y_*, *E_z_*)	200 GPa
Poisson's Ratio	0.29

**Table 2.** Natural frequencies of circular shaft.

**Mode**	**1st Bending**	**2nd Bending**	**3rd Bending**
Natural frequencies	770 Hz	2,159 Hz	4,235 Hz
Rotation speed equivalent	46,200 rpm	129,540 rpm	254,100 rpm

**Table 3.** Natural frequencies of circular shaft using ANSYS.

**Mode**	**1st Bending**	**2nd Bending**	**3rd Bending**
Natural frequencies	762 Hz	2,055 Hz	4,015 Hz
Rotation speed equivalent	46,500 rpm	127,380 rpm	252,900 rpm

**Table 4.** Specification test rig result.

**N_0_**	**Parameters**	**Values**
1	Measure range	±0.5 mm
2	DC output voltage resolution	2 mV/μm
3	Output voltage	−1 to 1 VDC
4	Linearity	±0.2% of full scale
5	Response frequency	DC to 4 kHz
6	Power supply	±9 VDC
